# Molecular Characterization and Antibiotic Resistant Profiles of *Campylobacter* Species Isolated From Poultry and Diarrheal Patients in Southeastern China 2017–2019

**DOI:** 10.3389/fmicb.2020.01244

**Published:** 2020-06-23

**Authors:** Leyi Zhang, Yi Li, Yongqiang Shao, Yuqin Hu, Huihuang Lou, Xiaonan Chen, Yuejin Wu, Lingling Mei, Biao Zhou, Xibin Zhang, Wenwu Yao, Lei Fang, Yanjun Zhang

**Affiliations:** ^1^Wenzhou Center for Disease Control and Prevention, Wenzhou, China; ^2^Ouhai Center for Disease Control and Prevention, Ouhai, China; ^3^Zhejiang Provincial Center for Disease Control and Prevention, Hangzhou, China; ^4^New Hope Liuhe Co., Ltd., Beijing, China

**Keywords:** *Campylobacter*, poultry, human feces, multidrug resistance, MLST, PFGE

## Abstract

*Campylobacter* is a zoonotic pathogen that causes foodborne diarrheal illness globally. To better understand health risks in Southeastern China, *Campylobacter* spp. were surveyed in humans and representative poultry products over 3 years. One hundred and ninety-five representative isolates (*n* = 148, *Campylobacter jejuni*; *n* = 45, *Campylobacter coli*; *n* = 2 *Campylobacter hyointestinalis*) were examined for genetic relatedness and antimicrobial susceptibility. Nearly all *Campylobacter* isolates (99.0%, 193/195) were resistant to at least one class of antimicrobials, and 45.6% (89/195) of the isolates exhibited multidrug resistance. Genotypic analysis revealed high diversity among tested strains. Multilocus sequence typing (MLST) displayed 120 sequence types (STs) including 42 novel STs being added to the PubMLST international database. Sixty-two STs belonged to 16 previously characterized clonal complexes (CCs), of which CC-21, CC-45, CC-464, CC-574, CC-353, and CC-828 were most frequently identified. In addition, pulsed-field gel electrophoresis (PFGE) fingerprinting resulted in 66 PFGE *Sma*I patterns among the 125 isolates, with eight patterns shared between human and poultry sources. Subtyping data did not correlate with antimicrobial resistance phenotypes. Taken together, this large-scale surveillance study highlights high antimicrobial resistance and molecular features of *Campylobacter* isolates in Southeastern China.

## Introduction

*Campylobacter* is a leading cause of sporadic bacterial gastroenteritis worldwide (Kirk et al., 2015; Li et al., 2018). In developed countries, *Campylobacter* infection is much more common than foodborne illness caused by *Listeria*, *Escherichia coli* O157, *Vibrio*, and *Shigella*, accounting for an estimated incidence of approximately 1.3 million foodborne cases annually in the United States alone (Scallan et al., 2011). *Campylobacter jejuni* and *Campylobacter coli* are the predominant cause of campylobacteriosis and responsible for approximately 95% of all *Campylobacter* infections (Coker et al., 2002). Campylobacteriosis is usually self-limiting and therefore does not require antimicrobial therapy. For patients with severe or prolonged symptomology, erythromycin or fluoroquinolone is the drug of choice (Butzler, 2004).

Poultry, particularly when handled improperly or consumed undercooked, has long been identified as the primary vehicle for sporadic campylobacteriosis in the United States and is the most common cause of *Campylobacter* outbreaks in Europe (Greig and Ravel, 2009; Zhao et al., 2010); however, the use of antimicrobials in food animals and their role in promoting antimicrobial resistance of *Campylobacter* to clinical important antibiotics remain controversial. Several studies showed that human infections with drug-resistant *Campylobacter* have increased in both developed and developing counties, coinciding with the introduction of antimicrobials for food animal production (Engberg et al., 2001; Kaakoush et al., 2015; Oh et al., 2017; Sproston et al., 2018; Woźniak-Biel et al., 2018). Indeed, drug-resistant *Campylobacter* has been classified as a serious antibiotic resistance threat in the United States. Antibiotic stewardship programs like the National Antimicrobial Resistance Monitoring System (NARMS) at CDC monitor antimicrobial resistance of foodborne pathogens and determine the source and magnitude of antimicrobial resistance from food supply, which in turn enable regulatory agencies to change management strategies. Conversely, an active surveillance system to monitor the prevalence and antimicrobial resistance of *Campylobacter* spp. in the food supply has not been established in China. Antibiotic stewardship is still challenging due to few regulations to control the use of antibiotics. In 2007, one *C. jejuni* outbreak was reported to be associated with the development of Guillain–Barrés syndrome in north China (Zhang et al., 2010). After that outbreak, Zhang et al. (2010) evaluated the genetic correlation of *C. jejuni* isolates from human patients and poultry in north China and found that all tested strains were resistant to nalidixic acid, levofloxacin, and ciprofloxacin, indicating drug resistance in clinical patients was likely linked with contaminated poultry products. However, as culture confirmation remains challenging, the link between food animals and human clinical isolates of *Campylobacter* has remained largely underreported and uncharacterized in China.

Therefore, the purposes of this study were to (i) investigate the genetic relatedness and linkage of *Campylobacter* strains from poultry products and human patients using pulsed-field gel electrophoresis (PFGE) and multilocus sequence typing (MLST) methods and (ii) determine the phenotypic antimicrobial resistance of these isolates in association with genotypic profiles and isolation sources in Southeastern China.

## Materials and Methods

### Sample Collection

One hundred and ninety representative poultry samples were collected periodically between 2017 and 2019 from farm markets (*n* = 106) in eight locations of Southeastern China. Representative poultry products consisted of chickens (*n* = 125), and ducks (*n* = 65) were kept fresh (never frozen) prior to sampling. During the sampling process, sterile containers were used to transfer representative poultry samples to Wenzhou CDC following aseptic practices. Eight hundred and fifty human stool samples (850 cases) were collected from foodborne diarrheal patients in two major hospitals of the greater Wenzhou area. Among the 850 cases, 462 were male and 388 were female. The age of the patients ranged from 6 months to 91 years. All samples were transported with ice packs and assessed within 4 h.

### Isolation and Species Identification

All samples were analyzed in triplicates for each date, site, and source. The prevalence of *Campylobacter* was evaluated as the percentage of positive samples from triplicate aliquots of all sample types. Bacterial isolation was performed by the enriched filtration method using *Campylobacter* isolation kits isolated (ZC-CAMPY-002, Qingdao Sinova Biotechnology Co., Ltd., Qingdao, China) according to the manufacturer’s instructions (Li et al., 2018). Presumptive positive colonies were streaked to Karmali agar and incubated for 48 h at 42°C in a microaerobic atmosphere (5% O_2_, 10% CO_2_, and 85% N_2_). Gram staining and biochemical identification including oxidase and catalase tests and hippurate hydrolysis were subsequently conducted. All putative *C. jejuni* and *C. coli* isolates from biochemical tests were subjected to multiplex PCR for species identification using species-specific primers as previously described (Klena et al., 2004). *Campylobacter hyointestinalis* samples were confirmed by 16S rRNA sequencing test. *C. jejuni* ATCC33560 provided by China Center of Industrial Culture Collection was used as the quality control strain.

### Multilocus Sequence Typing

Multilocus sequence typing was performed by the sequence analysis of seven housekeeping genes (*aspA*, *glnA*, *gltA*, *glyA*, *pgm*, *tkt*, and *uncA*) according to the protocol available on the PubMLST website^1^. Genomic DNA was extracted using Rapid Bacterial Genomic DNA Isolation Kit (NHUC004S, Novogene, Beijing, China) and amplified using the seven primer pairs as described previously (Dingle et al., 2001). Briefly, the amplification cycle was initial denaturation at 94°C for 5 min, followed by 35 cycles of 94°C for 30 s, 50°C for 30 s, 72°C for 45 s, and one final extension at 72°C for 5 min at the end of 35 cycles. Amplified DNA fragments were sequenced by Qinke Biotech Limited (Hangzhou, China). The nucleotide sequences for each locus were analyzed with BioNumerics software version 7.5 (Applied Maths, Belgium) and compared to published sequences on the PubMLST website. Sequence types (STs) were determined according the obtained seven-digit allelic profiles. A phylogenetic tree was generated using the unweighted pair-group method with arithmetic means (UPGMA) and the minimum spanning tree (MST) methods.

### Pulsed-Field Gel Electrophoresis Subtyping of *Campylobacter*

Pulsed-field gel electrophoresis was performed based on the CDC PulseNet protocol for *Campylobacter* spp. (Ribot et al., 2001). Briefly, DNA was digested with 40 U of *Sma*I enzyme (TaKaRa, Japan) at 30°C for 4 h. The separation of restriction fragments was performed in 1% SeaKem gold agarose (Lonza, Switzerland) gels in 0.5× Tris–borate–EDTA (TBE) buffer (Millipore Sigma, Burlington, MA, United States) using the CHEF Mapper system (Bio-Rad), with the following parameters: initial switch time, 6.76 s; final switch time, 35.38 s for 18 h at 6 V/cm, and condensation temperature of 14°C. PFGE profiles were analyzed using BioNumerics software version 7.5 (Applied Maths, Kortrijk, Belgium). *XbaI*-digested *Salmonella enterica* serovar Braenderup H9812 was used as a molecular size marker. The dendrogram was created by UPGMA with the Dice similarity coefficient and a position tolerance of 1.5%. Clusters were defined based on an 85% similarity cutoff.

### Antimicrobial Susceptibility Testing

The antibiotic susceptibility of 195 recovered *Campylobacter* isolates was determined using agar dilution method (M100-S25, 2015) against eleven antimicrobials, including azithromycin (AZI), ciprofloxacin (CIP), erythromycin (ERY), gentamicin (GEN), tetracycline (TET), florfenicol (FLO), chloramphenicol (CHL), streptomycin (STR), nalidixic acid (NAL), telithromycin (TEL), and clindamycin (CLI). They are classified into seven classes based on their antimicrobial mechanisms and importance for human campylobacteriosis treatment, including aminoglycosides (STR and GEN), quinolones (NAL and CIP), macrolides (ERY and AZI), lincosamides (CLI), tetracyclines (TET), phenicols (CHL and FLO), and ketolides (TEL). *C. jejuni* ATCC 33560 was used as a control organism. The susceptibility or resistance pattern of the *Campylobacter* isolates were interpreted as sensitive, intermediate, or resistant, according to the NARMS interpretive standards^2^ and epidemiological cutoff values as defined by the EUCAST^3^. The lowest MICs of the non-susceptible population were as follows: AZI, ≥8 μg/ml; CIP, ≥4 μg/ml; ERY, ≥32 μg/ml; GEN, ≥8 μg/ml; TET, ≥16 μg/ml; FLO, ≥8 μg/ml; CHL, ≥32 μg/ml; STR, ≥16 μg/ml; NAL, ≥64 μg/ml; TEL, ≥16 μg/ml; and CLI, ≥8 μg/ml. In addition, isolates exhibited resistant patterns to three or more groups of antibiotics which were considered as multidrug resistant (MDR).

### Statistical Analysis

The statistical software SPSS (Statistics 20, IBM, Armonk, NY, United States) was used for data processing and statistical analysis. A Chi-square test was used to analyze differences in the occurrence of positive samples between sources and species. Another Chi-square test was also performed to test for statistically significant associations between resistance to different antimicrobial drugs, different species, and *Campylobacter* isolates obtained from different sources. *P-*values of <0.05 were considered as significant. Simpson’s index (*D*) was assessed to determine the genetic diversity of *Campylobacter* subtypes (Hunter and Gaston, 1988). *D*-values closest to 1 indicate a high diversity, while *D*-values closest to 0 indicate a low diversity.

## Results

### Prevalence of *Campylobacter* spp. in Southeastern China

As shown in Table 1, *Campylobacter* strains were isolated from chicken (*n* = 80), duck (*n* = 26), and clinical samples (*n* = 89) between 2017 and 2019 at multiple sites in Southeastern China. The prevalence rate of *Campylobacter* was varied among different sources. 55.8% (106/190) of representative poultry products were contaminated with *Campylobacter*, which was significantly higher (*P* < 0.001) than those obtained in human stool samples (10.5%, 89/850). Among representative poultry products, a significant higher percentage (*P* = 0.002) of *Campylobacter* spp. isolates was observed in chicken samples (64%, 80/125) as compared to those in duck samples (40%, 26/65). Meanwhile, *C. jejuni* was recovered more frequently than either *C. coli* or *C. hyointestinalis* (*P* < 0.001). For instance, 36.3% (69/190) of representative poultry products and 9.3% (79/850) of clinical samples were positive for *C. jejuni*, whereas *C. coli* was only detected in 18.4% (35/190) and 1.2% (10/850) of these samples, respectively.

**TABLE 1:** Occurrence of *Campylobacter* species in poultry and clinical samples in Southeastern China.

**Sources**	**Number of sample**	**Prevalence of positive samples^1^ (%)**	**Species prevalence (positive sample)**		
			***C. jejuni* %**	***C. coli* %**	***C. hyointestinalis* %**
Chicken	125	64.0	43.2% (54)	20% (25)	0.8% (1)
Duck	65	40.0	23.1% (15)	15.4% (10)	1.5% (1)
Stool	850	10.5	9.3% (79)	1.2% (10)	0.0% (0)
Total poultry samples	190	55.8	36.3% (69)	18.4% (35)	1.1% (2)
Total samples	1040	18.8	14.2% (148)	4.3% (45)	0.2% (2)

*^1^Prevalence% = *n* positive/*n* tested.*

### MLST Analysis of *Campylobacter* Isolates From Various Sources

**FIGURE 1:**
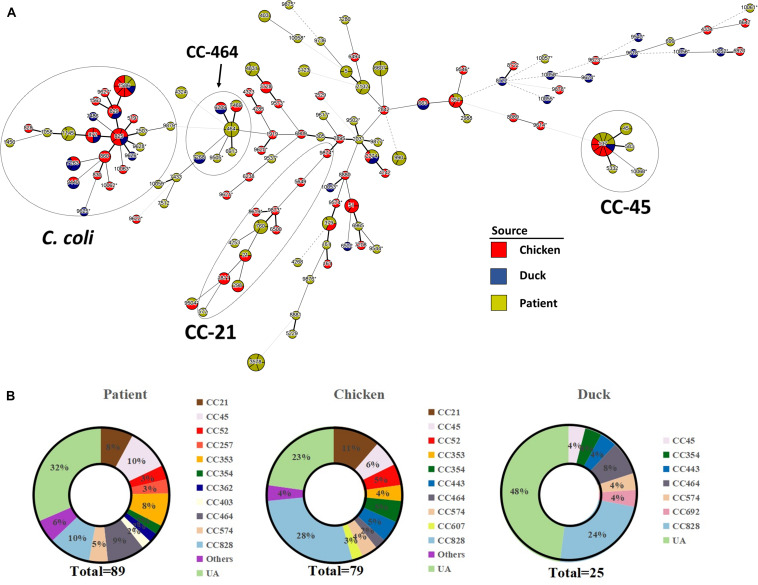
Clonal distribution analysis of *Campylobacter* spp. **(A)** Minimum spanning tree based on MLST data from 193 *Campylobacter* strains. Sources are distinguished by color differences (chicken, red; duck, blue; diarrheal patient, yellow). Different circles correspond to different STs. Size of circle indicates number of isolates within the same ST, and thickness of the branches represents the degree of similarity among *Campylobacter* tested. A bold solid line indicates that one allele is different according to the length. Two to three allele differences are represented by a thin solid line, and four is indicated by a wide-interval dotted line. A narrow-interval dotted line shows that there is a five-allele difference between STs. Nodes with fewer than three different alleles have been placed in the same cluster. Most STs in the circle of *C. coli* are assigned to CC-828, except ST-9625, ST-9440, ST-7263, and ST-1450. Asterisk indicates newly designated ST in this study. **(B)** Proportion of MLST clonal complexes in each source. UA, unassigned ST.

One hundred and ninety-three *Campylobacter* strains (except two *C. hyointestinalis* isolates) were further subjected to MLST analysis. An MST was generated from MLST data representing clonal distribution of *Campylobacter* strains from all three sources. Overall, 120 STs were overlaid onto the tree including 42 novel STs (16 STs from chicken, 10 STs from duck, and 16 STs from patient) and 62 STs which belonged to 16 previously characterized clonal complexes (CCs) (Figure 1A). Newly identified MLST profiles have been added to the *Campylobacter* PubMLST database (Supplementary Table 1). All *C. jejuni* isolates were classified into 97 different STs. Among them, 44 STs belonged to 15 previously characterized CCs, and the remaining 53 STs were unassignable to any CCs. *C. coli* strains clustered more closely as compared to *C. jejuni* strains. Forty-five *C. coli* isolates were divided into 23 distinct STs. CC-828 was the predominant clonal complex accounting for 88.3% (38/45) of all *C. coli* isolates, whereas the remaining seven isolates have STs that were unassigned.

The calculated Simpson’s index of *Campylobacter* isolates based on STs illustrated a high genetic diversity (*D* = 0.991). Three sequence types (ST-137, ST-1586, and ST-2274) were identified in all three sources (i.e., human clinical samples, chicken samples, duck samples). One ST (ST-7268) was identified only from duck products and human clinical cases, and six STs were identified only from chicken products and human clinical cases. Our results confirmed that CC-828 (19.7%, 38/193) was the major CC, followed by CC-21 (8.3%, 16/193), CC-45 (7.8%, 15/193), and CC-464 (6.2%, 12/193), regardless of source and species (Figure 1B). CC828, CC-45, and CC464 contained isolates from all three sources. CC-354 and CC-574 were also commonly found among different sources. CC-21, CC-52, and CC-353 were only associated with chicken and human clinical samples while CC-443 was poultry specific.

### PFGE Patterns of *Campylobacter* Isolates

**FIGURE 2:**
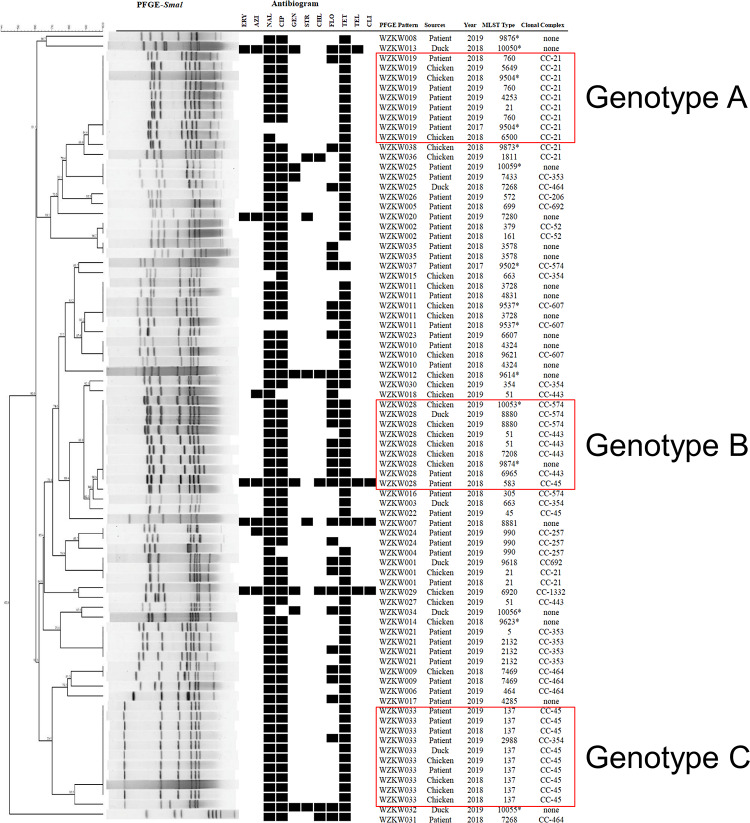
Dendrogram of 80 *C. jejuni* strains based on *Sma*I-mediated PFGE profiles. PFGE pattern, strain source, isolation year, sequence type, and clonal complex are depicted. Asterisk indicates newly designated ST in this study. Black squares of the antibiogram indicate resistance to antibiotics.

**FIGURE 3:**
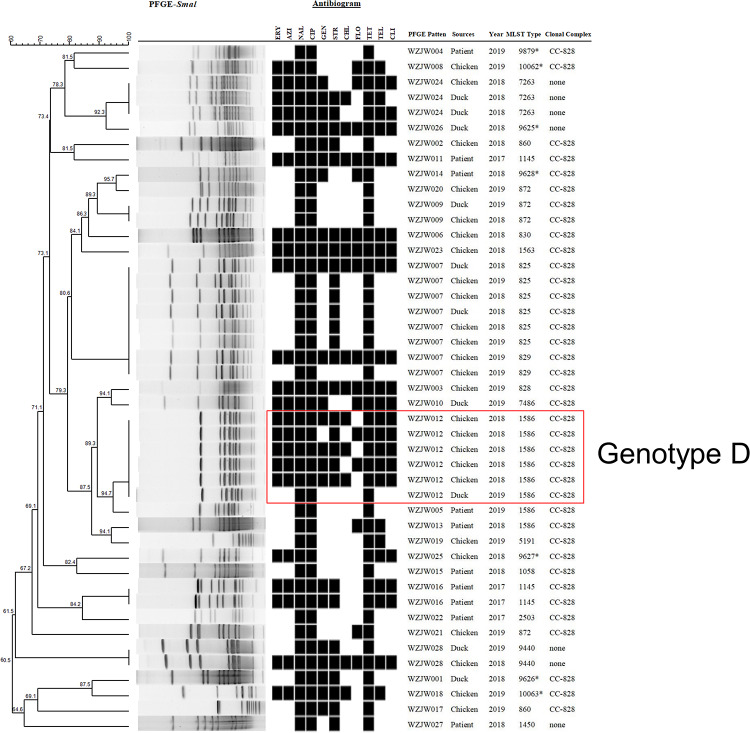
Dendrogram of 45 *C. coli* strains based on *Sma*I-mediated PFGE profiles. PFGE pattern, strain source, isolation year, sequence type, and clonal complex are depicted. Asterisk indicates newly designated ST in this study. Black squares in the antibiogram indicate resistance to antibiotics.

Pulsed-field gel electrophoresis was performed to assess the genetic relatedness of representative isolates (*n* = 125) from different sources, species, years, and MLST patterns. A high level of PFGE type diversity was observed among the 125 *Campylobacter* isolates (*D* = 0.975) as well as within each species (*D* = 0.954 and *D* = 0.951 for C. *jejuni* and *C. coli*, respectively). In total, the PFGE dendrogram contained 66 unique patterns; thirty-eight PFGE profiles were generated from 80 *C. jejuni* isolates (Figure 2) and 28 from 45 *C. coli* isolates (Figure 3). Of the 38 *C. jejuni* PFGE types, 17 and 13 distinct genotypes each were observed exclusively in human and representative poultry host populations, respectively, and eight genotypes were shared between the two host populations. In genotype C, five human isolates shared identical PFGE patterns with those from chicken (*n* = 4) and duck samples (*n* = 1). Likewise, two human isolates grouped together with the other seven from poultry host in genotype B. In respect to 45 *C. coli* isolates, representative poultry (*n* = 35) and human clinical isolates (*n* = 10) formed 19 and 9 pulsotypes, respectively (Figure 3). Thirty-eight *C. coli* isolates grouped to CC-828 were further divided into 23 unique PFGE types. Two human *C. coli* isolates (ST1586) clustered closely with chicken and duck isolates in genotype D (>87.5% similarity). However, no isolates from human formed identical PFGE patterns with chicken or duck among *C. coli* strains. The difference between the two species was expected because *C. coli* is less common as a cause of gastrointestinal infection than *C. coli.*

### Antibiotic Susceptibility Analysis

The 195 *Campylobacter* isolates were subjected to antimicrobial susceptibility tests against 11 antimicrobial agents belonging to seven different antimicrobial classes, and the frequency of antimicrobial resistant (AMR) results was presented in Table 2. Overall, 99.0% (193/195) of *Campylobacter* strains were resistant to at least one of the tested antimicrobials and displayed 56 different AMR patterns. Among the *C. jejuni* isolates, resistance to nalidixic acid (91.9%) was the most frequent, followed by resistance to ciprofloxacin (88.5%), tetracycline (87.2%), and florfenicol (46.6%). The rate of antimicrobial resistance was significantly higher in *C. coli* than that in *C. jejuni* strains (*P* < 0.001, χ^2^ = 28.907). All *C. coli* strains were resistant to nalidixic acid, ciprofloxacin, and tetracycline. Antibiotic resistance to streptomycin and gentamicin was also common for *C. coli* with resistance rates of 71.1 and 57.8%, respectively.

**TABLE 2:** Antimicrobial resistant rate of *Campylobacter* isolates from different species and sources against eleven antimicrobials.

**Rank^a^**	**Antimicrobial class**	**Antibiotic [breakpoints (μg/mL)]**	**Resistant rate (%) of isolates by species**		**Resistant rate (%) of isolates by sources**	
			***C. jejuni* (*n* = 148)**	***C. coli* (n = 45)**	**Poultry (*n* = 104)**	**Human (*n* = 89)**
I	Macrolides	ERY (≥32)	6.1	53.3	9.0	24.0
		AZI (≥8)	7.4	51.0	10.1	24.0
	Quinolones	NAL (≥64)	91.9	100.0	93.3	94.2
		CIP (≥4)	88.5	100.0	93.3	89.4
	Aminoglycosides	GEN (≥8)	12.2	57.8	12.4	31.7
		STR (≥16)	5.4	71.1	5.6	33.7
	Ketolides	TEL (≥16)	5.4	53.3	7.9	24.0
II	Tetracyclines	TET (≥16)	87.2	100.0	86.5	93.3
	Lincosamides	CLI (≥8)	5.4	42.2	7.9	19.2
	Phenicols	CHL (≥32)	7.4	28.9	5.6	18.3
		FLO (≥8)	46.6	40.0	45.0	45.2

*^*a*^Rank of antimicrobial agents is based on World Health Organization’s categorization of critical importance in human drugs. Rank I, critically important; rank II, highly important.*

Approximately 74% (142/193) *Campylobacter* strains were categorized into fourteen major AMR profiles (Table 3). To be noted, 45.1% (88/195) of the isolates exhibited multidrug resistance (MDR, resistance to three or more classes of antibiotics), including five strains that were resistant to ten antimicrobials and ten strains that were resistant to all tested antimicrobials. Multidrug-resistant poultry isolates were common. Representative poultry isolates (61.3%, 65/106) exhibited a significantly higher rate of MDR (*P* = 0.048, χ^2^ = 3.901) compared to those isolates collected from human fecal samples (47.2%, 42/89). NAL-CIP-TET was the dominant AMR profile for both poultry and human samples presenting AMR rates of 25.5% (27/106) and 36.0% (32/89), respectively.

**TABLE 3:** Major antimicrobial resistance patterns of *C. jejuni* and *C. coli*.

**Antimicrobial resistance profile (*n* = agents)**	**AMR prevalence (%) of isolates by species**	
	***C. jejuni* (*n* = 148)**	***C. coli* (*n* = 45)**
ERY-AZI-NAL-CIP-GEN-STR-CHL-FLO-TET-TEL-CLI^b^ (11)	1.4	17.8
ERY-AZI-NAL-CIP-GEN-STR-CHL-TET-TEL-CLI^b^ (10)	0.0	6.7
ERY-AZI-NAL-CIP-GEN-CHL-FLO-TET-TEL-CLI^b^ (10)	1.4	0.0
ERY-AZI-NAL-CIP-GEN-STR-CHL-TET-TEL^b^ (9)	0.0	4.4
ERY-AZI-NAL-CIP-GEN-STR-TET-TEL-CLI^b^ (9)	0.0	6.7
NAL-CIP-GEN-STR-TET^b^ (5)	0.0	8.9
NAL-CIP-GEN-FLO-TET^b^ (5)	0.7	2.2
NAL-CIP-FLO-CHL-TET^b^ (5)	1.4	0.0
NAL-CIP-GEN-TET^b^ (4)	1.4	0.0
NAL-CIP-STR-TET^b^ (4)	0.7	13.3
NAL-CIP-FLO-TET^b^ (4)	27.0	0.0
NAL-CIP-FLO (3)	3.4	0.0
NAL-CIP-TET (3)	31.8	20.0
NAL-CIP (2)	2.7	0.0
Others	18.2	20.0
MDR%^a^	34.5	80.0

*^*a*^Prevalence of multidrug-resistant isolates in *C. jejuni* or *C. coli*. ^*b*^AMR profile exhibited MDR characteristic.*

Considering resistance by species, 34.5% of *C. jejuni* strains were MDR and the dominant MDR pattern was NAL-CIP-FLO-TET (27.0%). *C. coli* strains (80.0%) displayed significantly higher MDR rates (*P* < 0.001) than did *C. jejuni*. The primary MDR pattern for *C. coli* strains was ERY-AZI-NAL-CIP-GEN-STR-CHL-FLO-TET-TEL-CLI (17.8%). Notably, the strains that showed resistant to eleven agents were exclusively confirmed as *C. coli*. With respect to two *C. hyointestinalis* isolates, both were isolated from retail poultry meats and one isolate showed to be resistant to CIP-TET, while the other one exhibited an MDR feature (ERY-AZI-NAL-CIP-GEN-STR-FLO-TET-TEL).

Subtyping data did not correlate with antimicrobial resistance phenotypes. All CCs contained both resistant and susceptible populations. A similar situation was seen in two major genotypes (B and C). All *C. jejuni* strains assigned to genotype B and genotype C, respectively, were resistant to the NAL + CIP + TET profile. No specific association was noted between isolation year and AMR phenotype as well, demonstrating that antibiotic non-susceptibility may evolve slowly in our study region.

## Discussion

To quantify *Campylobacter* diversity and aid in controlling *Campylobacter*-borne infection, we characterized the distribution, subtype diversity, and antimicrobial susceptibility of 195 *Campylobacter* isolates collected from representative poultry products and humans in Southeastern China. A total of 55.8% of representative poultry samples were contaminated with *Campylobacter*, which was higher than that detected in other regions of China (Huang et al., 2009; Ma et al., 2017; Zhang et al., 2017). Chickens (64%) were identified as the major source of infection in the study area, while the contamination rate of *Campylobacter* spp. in ducks (40%) was found at a clearly lesser extent. These results were consistent with reports from many developed countries, where chicken was a predominant reservoir of *Campylobacter* spp. and was also recognized as potential vehicle of infection in humans (Stern et al., 2001; Strachan et al., 2012, 2013; Szczepanska et al., 2017). The presence of *C. hyointestinalis* strains from representative poultry isolates was worth mentioning, indicting species diversity of *Campylobacter* spp. in poultry population hosts.

A high prevalence of *Campylobacter* infection (10.5%) in diarrheal patients contrasts with a prior report that no campylobacteriosis cases were observed in Northern China (Wang et al., 2015). The increased incidence of *Campylobacter* illness in our study may be due to the increased recognition and enhanced surveillance of this pathogen rather than the absence of illness related to this microorganism. The application of the novel filtration method in our study may also facilitate detection of *Campylobacter* from human stool samples (Li et al., 2018; Liang et al., 2018).

Consistent with previous reports, the MLST data confirmed that *Campylobacter* is genetically diverse, with *C. jejuni* isolates being more diverse than *C. coli* (Kinana et al., 2007; Wang et al., 2011; Ngulukun et al., 2016). The dominant CCs in this study were CC-21 and CC-45 and were in agreement with prior findings from other countries in which CC-21 and CC-45 appear to be the largest complexes (Sheppard et al., 2010; Guyard-Nicodème et al., 2015; An et al., 2018). The prevalence of CC-353, CC-464, and CC-828 was lower than those in other regions of China (Zhang et al., 2017), supporting the idea that *Campylobacter* subtype diversity may differ by region and sampled area. The association between CCs and hosts was previously noted and was reaffirmed by the present survey. The presence of CC-45 in chickens, ducks, and humans was consistent with previous studies showing that CC-45 exhibited “multi-host” characteristics and have been isolated from environmental and agricultural sources (Colles and Maiden, 2012; Guyard-Nicodème et al., 2015). The identification of CC-45 in humans (*n* = 4) was highly noteworthy in this survey, as it has been proposed as a specific clonal linage linked with Guillain–Barrés syndrome (Zhang et al., 2010). CC-21 was only found from human and chicken sources but not duck samples, which differed from an analysis of ducks isolated from South Korea (Wei et al., 2014). Likewise, CC-353 was commonly associated with food and human isolates in other regions of China (Zhang et al., 2017), while it was only found in the chicken and human sources in our study. These findings lend weight to the suggestion that specific CCs of *Campylobacter* might be associated with certain regions.

Pulsed-field gel electrophoresis was superior in discriminating clonal strains from multiple sources for tracking the primary origin. CC-353, CC464, CC-574, and CC-828 exhibited diverse PFGE patterns, indicating a weak correlation between PFGE and MLST (Figures 2, 3). However, some agreement between PFGE and MLST was observed for certain STs or clonal complex. For example, all isolates belonging to ST-137 (CC-45) were grouped together in genotype B. It was observed that human strains exhibited patterns that were indistinguishable from chicken and duck strains within CC-45, indicating that strains from diverse sources may be transmitted through complex routes between farm animals, production environment, and humans. In genotype A, all CC-21 isolates from chicken hosts were clustered together with human strains, highlighting the important role of chicken products in human campylobacteriosis. Since campylobacteriosis is transmitted primarily through food, these data underscored the necessity to enhance food safety management in an animal production environment to prevent contamination routes associated with different hosts and regions. Our findings also demonstrated the usefulness of accompanying MLST with PFGE to facilitate the source tracking of *Campylobacter* as *Campylobacter* subtypes can be highly associated with certain hosts.

An important issue in public health is the emergence of antibiotic-resistant strains of *Campylobacter*. Overall, *C. coli* showed higher resistance rates to all antimicrobials than *C. jejuni* did except for florfenicol. It is worth mentioning that 43.0% (34/79) and 50.7% (35/69) of *C. jejuni* from patients and poultry samples were resistant to florfenicol in this study, which were much higher than those in previous reports with a prevalence rate less than 10% (Tang et al., 2017; Zhao et al., 2019). Florfenicol is only permitted for veterinary use. The paralleled rise of FLO^r^ *Campylobacter* infection in humans and food-producing animals suggests the potential transmission of resistance genes via the foodborne route. In addition, 94.9% (75/79) of *C. jejuni* clinical samples were susceptible to erythromycin, indicating that erythromycin remains effective for the treatment of *C. jejuni* infection in Southeastern China.

*Campylobacter jejuni* was mainly resistant to nalidixic acid (91.9%), ciprofloxacin (88.5%), and tetracycline (87.2%), while all *C. coli* isolates were resistant to these three antibiotics. Similar observations have been reported from poultry and human host populations in Central (Zhang et al., 2016) and North China (Zhang et al., 2010), as well as other countries (Han et al., 2007; Rahimi et al., 2010). High-level resistance to ciprofloxacin is problematic clinically as treatment failure could have significant consequences for immunocompromised patients. An alarming situation was noted in this study. Similar resistance phenotypes and PFGE patterns were observed among CIP^r^ *Campylobacter* isolates from human and poultry sources, indicating that the human isolates were likely linked to the contaminated poultry products. Anthropogenic impact on bacteria in poultry production, such as the approval of fluoroquinolone use in animal husbandry, may contribute to the selection of CIP^r^ *Campylobacter* strains that are transmitted to humans through the food chain in the tested region. Future studies are needed to fill the gap between animal use antibiotic and ciprofloxacin resistance in human infections.

To the best of our knowledge, this is the first report describing the genetic distribution and antibiotic resistance features of *Campylobacter* spp. from retail poultry meats and humans in Southeastern China. The MLST distribution of *Campylobacter* spp. showed to be clonal among *Campylobacter* isolates, and chicken was considered as the major reservoir of *Campylobacter* infection. High PFGE diversity was observed in three different sources, and some human isolates were indistinguishable and/or highly related with poultry isolates. Although the composite analysis of MLST and PFGE enhanced the resolution potential of strain subtyping, WGS-based typing has been progressively replacing traditional typing methods. Future work is warranted to integrate WGS into routine monitoring as the primary typing method for *Campylobacter* detection and epidemiologic investigations. In addition, our results confirmed that most *Campylobacter* isolates from different sources were resistant to at least one of the eleven antimicrobials tested and MDR was common. The resistance to ciprofloxacin was highly undesirable. These results underscored the necessity for better understanding of the mechanisms that drive antibiotic resistance in *Campylobacter* and more rigorous surveillance to tracking antimicrobial resistance in epidemiologically distinct populations.

## Data Availability Statement

The raw data supporting the conclusions of this article will be made available by the authors, without undue reservation, to any qualified researcher.

## Ethics Statement

The studies involving animals were reviewed and approved by the Zhejiang Provincial Center for Disease Control and Prevention Ethics committee. The studies involving human participants were also reviewed and approved by the Zhejiang Provincial Center for Disease Control and Prevention Ethics committee. The patients/participants provided their written informed consent to participate in this study.

## Author Contributions

LZ, YL, and YS conceived and designed the study. YH, HL, XC, and YW performed the sampling. LF, YL, and LZ analyzed MLST and PFGE data, supervised the study, and revised the manuscript. LF and LZ prepared the manuscript. LM and YZ performed the administration. WY, XZ, BZ, and YZ contributed reagents and, materials and analysis tools. All authors contributed to the article and approved the submitted version.

## Conflict of Interest

XZ employed by New Hope Liuhe Co., Ltd. The remaining authors declare that the research was conducted in the absence of any commercial or financial relationships that could be construed as a potential conflict of interest.

## Footnotes

1 http://pubmlst.org/campylobacter/

2 https://www.cdc.gov/narms/antibiotics-tested.html

3 http://www.eucast.org/
