# Supplementary material for: Molecular Characterization and Antibiotic Resistant Profiles of Campylobacter Species Isolated From Poultry and Diarrheal Patients in Southeastern China 2017–2019
Source: Front Microbiol. 2020 Jun 23;11:1244. doi: 10.3389/fmicb.2020.01244 (PMC7324532; doi:10.3389/fmicb.2020.01244)
Supplement: Supplementary file 1 [file Data_Sheet_1.PDF]

Supplementary Table. MLST data summary of 41 novel STs

Strain	MLST									Source	Isolation Year	Species
	aspA	*glnA*	*gltA*	*glyA*	*pgm*	*tkt*	*uncA*	ST	Clonal			
1808002	2	53	5	10	11	3	3	9874*	ST-574	Chicken	2018	*Campylobacter jejuni*
1808008	2	17	12	3	11	1	6	9504*	none	Chicken	2018	*Campylobacter jejuni*
1808009	8	61	57	28	470	29	5	9542*	none	Duck	2018	*Campylobacter jejuni*
1808017	2	1	52	3	11	100	5	9873*	ST-21	Chicken	2018	*Campylobacter jejuni*
1808031	8	17	2	2	2	12	6	9546*	ST-354	Chicken	2018	*Campylobacter jejuni*
1808037	9	2	1	10	17	3	1	9573*	none	Chicken	2018	*Campylobacter jejuni*
1808044	14	21	2	10	11	3	12	9574*	ST-574	Chicken	2018	*Campylobacter jejuni*
1808047	304	39	30	79	104	206	325	9625*	none	Duck	2018	*Campylobacter coli*
1808048	4	455	291	668	127	24	1	9614*	none	Chicken	2018	*Campylobacter jejuni*
1808049	33	491	30	82	113	47	17	9626*	ST-828	Duck	2018	*Campylobacter coli*
1808051	33	39	30	82	113	43	139	9627*	ST-828	Chicken	2018	*Campylobacter coli*
1808059	9	52	57	26	74	25	23	9618*	ST-692	Duck	2018	*Campylobacter jejuni*
1808063	14	1	52	3	11	100	5	9619*	none	Chicken	2018	*Campylobacter jejuni*
1808065	24	23	12	496	23	25	6	9624*	none	Duck	2018	*Campylobacter jejuni*
1808066	8	61	12	496	188	25	6	10050*	none	Duck	2018	*Campylobacter jejuni*
1808067	8	61	57	26	74	25	271	9623*	none	Chicken	2018	*Campylobacter jejuni*
1808068	7	438	292	2	13	25	23	9622*	none	Chicken	2018	*Campylobacter jejuni*
1808070	9	2	2	10	22	3	5	9620*	ST-52	Chicken	2018	*Campylobacter jejuni*
1808071	8	2	27	751	22	3	1	9621*	ST-607	Chicken	2018	*Campylobacter jejuni*
1908009	37	61	4	64	982	7	6	9878*	none	Chicken	2019	*Campylobacter jejuni*
1908019	32	39	30	79	113	47	139	10062*	none	Chicken	2019	*Campylobacter coli*
1908024	2	486	4	28	129	25	23	10052*	none	Duck	2019	*Campylobacter jejuni*
1908031	7	17	2	10	11	25	12	10053*	ST-574	Duck	2019	*Campylobacter jejuni*
1908035	8	59	29	64	470	407	1	10055*	none	Duck	2019	*Campylobacter jejuni*
1908037	8	52	225	28	129	25	23	10056*	none	Duck	2019	*Campylobacter jejuni*
1908041	33	42	30	82	113	35	17	10063*	none	Chicken	2019	*Campylobacter coli*
B1708004	9	17	12	10	11	3	3	9502*	ST-574	Patient	2017	*Campylobacter jejuni*
B1708008	2	17	12	3	11	1	6	9504*	none	Patient	2017	*Campylobacter jejuni*
B1708009	24	2	52	2	10	1	1	9505*	ST-464	Patient	2017	*Campylobacter jejuni*
B1808021	8	2	27	10	11	3	5	9537*	ST-607	Patient	2018	*Campylobacter jejuni*
B1808026	7	2	2	15	10	3	3	9544*	ST-574	Patient	2018	*Campylobacter jejuni*
B1808039	33	39	30	82	113	12	17	9628*	ST-828	Patient	2018	*Campylobacter coli*
B1808045	7	53	5	10	2	3	3	9617*	ST-574	Patient	2018	*Campylobacter jejuni*
B1808047	37	2	5	64	332	668	1	9875*	none	Patient	2018	*Campylobacter jejuni*
B1908001	8	2	2	10	86	3	147	9876*	none	Patient	2019	*Campylobacter jejuni*
B1908007	9	17	5	10	74	3	3	9877*	none	Patient	2019	*Campylobacter jejuni*
B1908008	33	2	30	82	104	3	17	9879*	ST-828	Patient	2019	*Campylobacter coli*
B1908016	8	364	2	64	694	99	3	10057*	none	Patient	2019	*Campylobacter jejuni*
B1908026	7	592	59	19	10	5	7	10058*	none	Patient	2019	*Campylobacter jejuni*
B1908027	7	17	358	2	22	3	6	10059*	none	Patient	2019	*Campylobacter jejuni*
B1908033	4	52	4	4	42	25	1	10060*	none	Patient	2019	*Campylobacter jejuni*
B1908036	8	348	4	26	74	546	23	10061*	none	Patient	2019	*Campylobacter jejuni*
